# Infection au virus Zika: transmission sexuelle et conséquences sur la prévention: Présentation faite lors de la Journée scientifique de la SFMTSI du 9/11/2023 « Infections sexuellement transmissibles tropicales »

**DOI:** 10.48327/mtsi.v4i2.2024.502

**Published:** 2024-04-02

**Authors:** Paul Henri CONSIGNY

**Affiliations:** Centre médical de l’Institut Pasteur, 211 rue de Vaugirard, 75015 Paris, France

**Keywords:** Virus Zika, Transmission sexuelle, Sperme, Sécrétion vaginale, Prévention, Voyage, Zika virus, Sexual transmission, Semen, Vaginal secretion, Prevention, Travel

## Abstract

L’infection au virus Zika, responsable le plus souvent d’un tableau arboviral bénin ou d’une infection asymptomatique, peut devenir problématique chez les femmes enceintes, en raison d’un risque de malformations fœtales. La transmission est majoritairement vectorielle, mais une transmission sexuelle est décrite, principalement chez des voyageurs, même si elle représente sans doute une part significative de la transmission en zone épidémique. Cette revue vise à décrire cette transmission sexuelle au travers principalement des exemples liés à l’épidémie de grande ampleur survenue en 2016 en Amérique latine, à en décrire le lien avec l’excrétion prolongée de particules virales infectieuses dans les sécrétions génitales, et à mettre en exergue les mesures de prévention possibles en dehors de la transmission vectorielle.

## Abstract (en)

Zika virus infection, most oft n responsible for a benign arboviral disease or an asymptomatic infection, rarely Guillain-Barré syndrome, can become problematic in pregnant women, due to a risk of fetal malformations, in particular microcephaly linked to its neurotropism. The most recent large-scale epidemic was observed throughout Latin America between 2015 and 2017, causing several hundred thousand cases. Transmission is predominantly vector-borne, but sexual transmission has been described, mainly among travelers, although it undoubtedly accounts for a significant proportion of transmission in epidemic areas. The aim of this review is to describe this sexual transmission, mainly through examples linked to this large-scale epidemic in Latin America, to describe the link with prolonged excretion of infectious viral particles in genital secretions, especially semen but also vaginal secretions, and to highlight possible preventive measures apart from vector transmission, in particular the need for pregnant women or women wishing to become pregnant to avoid visiting countries where circulation of Zika virus is described.

## L'infection au virus Zika : epidemiologie, clinique, complications

Le virus Zika est un arbovirus de la famille des Flaviviridae, du genre *Flavivirus,* qui a été isolé pour la première fois chez un macaque sentinelle en 1947, dans la forêt de Zika en Ouganda. La date du premier cas humain, observé entre 1954 au Nigéria et 1963 en Ouganda, reste controversée. Le cycle naturel du virus est selvatique, entre des moustiques vecteurs du genre *Aedes,* sous-genre *Stegomyia* ou *Diceromyia,* et des primates non humains, à l’origine de cas humains sporadiques. Les épidémies en milieu urbain ou péri-urbain sont plutôt liées à *Aedes (Stegomyia) aegypti* ou *Aedes (Stegomyia) albopictus,* ce dernier ayant une part moins prépondérante dans la transmission vectorielle que l’Aedes *aegypti* [28].

**Figure 1:**
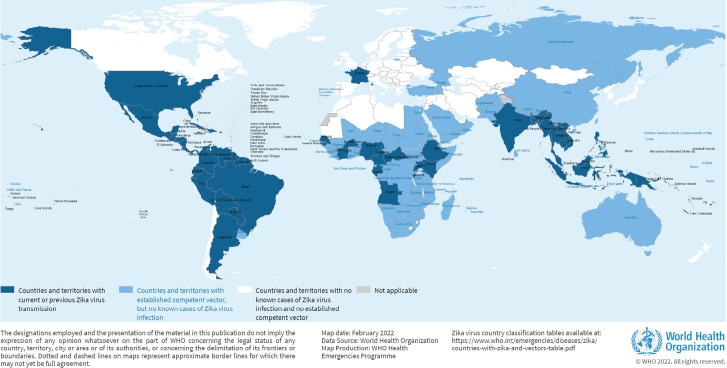
Pays et territoires avec circulation présente ou passée du virus Zika (© OMS 2022) Countries and territories with current or previous Zika virus circulation (© WHO 2022)

Depuis sa première description chez l’humain, plusieurs épidémies d’ampleur limitée ont été observées en Afrique sub-saharienne (République centrafricaine, Sénégal, Burkina Faso, Côte d’Ivoire, Cameroun, Sierra Léone, Gabon, Nigéria), puis en Asie (Pakistan, Indonésie, Malaisie, Cambodge), avant une première épidémie de grande ampleur sur l’île de Yap, états fédérés de Micronésie, en 2007, avec 5 000 cas, correspondant à un taux d’attaque de 75 % de la population totale [2, 34]. La 2^e^ épidémie de grande ampleur est survenue en Polynésie Française en 2013, avec près de 30 000 cas estimés, pour un taux d’attaque de 11,5 % de la population polynésienne, d’autres zones d’Asie ou du Pacifique étant atteintes en parallèle entre 2012 et 2015 (Îles Cook, Nouvelle-Calédonie, Vanuatu, Fidji, Samoa, Îles Solomon, Philippines, Cambodge, Indonésie, Thaïlande, île de Pâques) [2, 34]. C’est d’ailleurs à l’occasion de cette épidémie de grande ampleur qu’ont été décrites les premières formes compliquées liées au virus Zika, formes neurologiques à type de syndromes de Guillain-Barré [4]. Mais c’est l’année 2015 qui marque une étape importante dans la progression épidémiologique du virus Zika avec son arrivée et sa diffusion rapide sur le continent américain, en particulier sud-américain. Les premiers cas au Brésil sont décrits en mai 2015 dans l’état du Nordeste, suivis d’une croissance rapide du nombre de cas, aboutissant à plus de 220 000 cas jusqu’en mai 2017, et surtout aux premières descriptions des embryo-fœtopathies liées à l’infection congénitale au virus Zika, en particulier les premiers cas de microcéphalies [2]. Durant les années 2015 à 2017, une extension rapide de l’épidémie a été observée. Quasiment tous les États et territoires d’Amérique du Sud et d’Amérique centrale ont été touchés, 52 au total, dont une grande partie des îles des Antilles, pour un total estimé à près de 800 000 cas [25]. L’épidémie s’est terminée courant 2018 dans la plupart des pays et territoires, avec un arrêt ou une très nette régression du nombre de cas. Seul le Brésil a vu persister un nombre significatif de contaminations depuis, avec plus de 60 000 cas en 2022-2023. En dehors de l’Amérique latine, où des cas ont pu aussi être observés dans les pays andins ou en Amérique centrale, des cas sporadiques ont été déclarés sur d'autres continents. Ainsi, deux cas autochtones ont été rapportés en France métropolitaine en 2019 dans le Var [15]. En 2021, une circulation virale a été mise en évidence en Inde dans plusieurs États du Sud et du Centre, après un diagnostic de Zika chez une femme enceinte, qui a permis de diagnostiquer *a posteriori* plusieurs cas parmi des syndromes éruptifs ou sur des prélèvements sanguins effectués à cette période [35]. Enfin, en 2022 en Thaïlande, un nombre significatif de touristes ont été touchés, le diagnostic étant posé à leur retour dans leur pays d’origine [31]. Ces épisodes de circulation témoignent de la difficulté de mettre en évidence une circulation à bas bruit de ce virus en dehors des épidémies de grande ampleur. La situation actuelle est à ce titre difficile à caractériser au plan de la circulation virale réelle : la dernière carte OMS disponible rapporte les pays ayant ou ayant eu une circulation virale autochtone « non silencieuse » du virus Zika (Fig. 1).

Au plan clinique, après un délai d’incubation de 3 à 12 jours, l’infection au virus Zika se manifeste par un syndrome arboviral sans gravité, associant classiquement une éruption cutanée, une conjonctivite, des arthromyalgies, des céphalées et une fièvre inconstante [23].

Le tableau clinique est cependant asymptomatique dans la majorité des cas, entre 50 et 80 %. Les complications sont principalement le fruit de son neurotropisme, avec des syndromes de Guillain-Barré (d’incidence estimée à 0,24/1 000 lors de l’épidémie de 2014 en Polynésie française [4]), parfois des myélites ou des méningo-encéphalites, des uvéites, et plus rarement des complications extra-neurologiques. Dans le cas général, l’évolution se fait favorablement en une semaine. La létalité est exceptionnelle, inférieure à 0,01 %.

Mais la complication désormais la plus emblématique de cette infection est liée à la transmission materno-fœtale, possible tout au long de la grossesse, responsable du syndrome de Zika congénital, dont la gravité est d’autant plus fréquente en cas d’infection en début de grossesse, au 1^er^ trimestre, avec la possibilité de microcéphalies [23]. Cette transmission verticale avec infection fœtale concernerait 20 à 30 % des grossesses, résultant, sur la totalité des grossesses, en 4 à 7 % de pertes fœtales et 5 à 14 % d’anomalies neurologiques congénitales, dont 4 à 6 % de microcéphalies [23]. Il semblerait cependant que ces données soient un peu surestimées, si l’on en croit les données observées à l’issue de l’épidémie de Zika en Guadeloupe en 2016-2017 : alors que le risque de Zika congénital y était estimé à 7 % (dont 12,7 % au 1^er^ trimestre et 3,6 à 5,3 % aux 2^e^ et 3^e^ trimestre), une comparaison de la proportion d’anomalies morphologiques constatées à la naissance chez les enfants nés de mères infectées ou non par le virus Zika pendant leur grossesse a permis de réestimer la part attribuable au virus Zika, et de réévaluer le risque global de Zika congénital à 1,6 %, représentant respectivement des risques de 4,1 %, 0,8 % et 0 % aux 1^er^, 2^e^ et 3^e^ trimestre [14, 19]. Ces complications ont pu être observées quel que soit le statut symptomatique (ou pas) de la mère.

## La transmission sexuelle du virus Zika

Le principal mode de transmission du virus Zika reste d’ordre vectoriel, le repas sanguin du moustique se faisant sur un individu infecté et virémique, soit entre 1 et 2 jours avant le début des symptômes et 7 jours après, mais d’autres modes de transmission ont été mis en évidence. La transmission sexuelle en constitue le principal mode alternatif, avec la transmission materno-fœtale transplacentaire, les autres modes étant bien plus occasionnels, comme la transmission par transfusion ou par le biais d’une transplantation d’organe ou de moelle, ou par accident d’exposition au sang en laboratoire. Par contre, à ce jour, l’allaitement ne constitue pas un mode de transmission reconnu.

S’il a fallu attendre l’épidémie de grande ampleur de 2016 en Amérique latine pour obtenir la preuve définitive de cette transmission sexuelle, quelques données antérieures permettaient déjà de la suspecter. Une première transmission non vectorielle du virus Zika avait pu être observée en 2008 aux USA dans le Colorado, avec une infection symptomatique rapportée chez une femme 4 jours après son mari, un scientifique ayant lui-même présenté un Zika symptomatique 6 jours après son retour du Sud-Est du Sénégal. L’épouse n’avait pas voyagé et l’analyse des moustiques autochtones n’avait pu mettre en évidence de virus Zika localement. Le diagnostic ayant été sérologique, aucune preuve moléculaire n’a pu être obtenue chez les deux personnes du couple [11]. Ensuite, à l’occasion de l’épidémie de 2014 en Polynésie française, du virus Zika avait été mis en évidence chez un homme ayant consulté pour hémospermie et ayant présenté un syndrome grippal plus de 15 jours avant, cette mise en évidence l’ayant été par PCR spécifique dans le sperme et par culture virale, confirmant l’infectiosité du prélèvement de sperme, même si dans ce cas, aucune transmission n’a été prouvée chez un ou une partenaire [24]. En outre, la quantité de virus dans le sperme était très importante, atteignant 10^7^ copies, alors qu’elle n’était pas détectable dans le sang en parallèle.

C’est donc à l’occasion de la grande épidémie de 2016 qu’a pu être documentée de façon formelle la transmission sexuelle, comme dans le cas rapporté en 2016 par d’Ortenzio [10] : dans cette observation clinique, un voyageur a contracté le Zika à l’occasion d’un séjour au Brésil, avec un tableau symptomatique ayant débuté 3 jours avant le retour en France, suivi 9 jours après son retour d’un même tableau symptomatique chez sa compagne qui n’avait pas voyagé. Le couple a eu des rapports sexuels dès le retour du Brésil. La preuve de la transmission a été apportée par la comparaison phylogénétique des séquences issues des virus isolés (par PCR et culture) dans la salive de la patiente (à J3 de son tableau clinique) et dans le sperme du patient (à J18 de son tableau clinique, correspondant au J5 de sa partenaire), alors même que les prélèvements sanguin et salivaire étaient négatifs chez lui. Il est à noter que le prélèvement de sperme restera positif chez l’homme jusqu’à J80, avant négativation à J93 [20].

À la même période, de multiples descriptions ont été faites, presque exclusivement chez des conjointes de voyageurs de retour de zone épidémique, mais n’ayant pas voyagé, principalement dans des pays d’Europe ou d’Amérique du Nord [12,18,33]. La preuve d’une transmission non vectorielle est en effet bien plus difficile à apporter en zone d’épidémie active de Zika. Ces descriptions ont inclus des cas de survenue tardive par rapport au retour de voyage et au tableau symptomatique du cas index, comme dans l’observation décrite par Turmel. Ce dernier relate la survenue chez une femme d’un tableau symptomatique 44 jours après le début du tableau clinique de son conjoint, en faisant une transmission sexuelle entre 32 et 41 jours après le tableau initial chez l’homme, soit le délai le plus important décrit dans une transmission sexuelle [32]. Elles ont aussi inclus un cas de transmission depuis un homme qui avait subi une vasectomie, indiquant le caractère potentiellement infectieux des différentes sécrétions tant séminales que pré-séminales [1].

Le caractère symptomatique du patient index n’est absolument pas impératif pour assurer une transmission sexuelle, même s’il est le plus souvent rapporté dans les différentes observations, car de telles transmissions ont pu être documentées chez des patientes dont le conjoint était asymptomatique mais ayant fait la preuve d’une infection et présentant une PCR positive dans le sperme [3, 13].

Des transmissions sexuelles ont aussi pu être décrites entre deux hommes, après un rapport anal actif [8] ou d’une femme vers un homme [7]. Dans ce dernier cas, la transmission est suspectée être d’origine vaginale, avec un rapport sexuel 24h avant le début du tableau symptomatique, 4 jours avant une documentation de virémie chez la patiente et 9 jours avant chez le patient. Sur une revue des 27 premières observations de transmission sexuelle, 25 étaient d’un homme vers une femme, 1 d’un homme vers un homme et 1 d’une femme vers un homme [22]. Les rapports sexuels rapportés étaient vaginaux dans 96 % des cas, oraux dans 18 % des cas et anaux dans 7 % des cas [22].

L’explication de cette « efficacité » bien supérieure de la transmission de l’homme vers la femme que de la femme vers l’homme, tient à la persistance virale dans les sécrétions, bien plus importante dans le sperme que dans les sécrétions vaginales. La cinétique de persistance du virus Zika dans le sperme a pu être analysée chez 184 volontaires américains ayant présenté un Zika symptomatique : au total, un tiers des patients a présenté un prélèvement de sperme positif par PCR sur la période de suivi, le prélèvement positif le plus tardif ayant été recueilli 281 jours après le début de l’infection au virus Zika (contre un maximum de 91 jours pour la salive ou l’urine et 34 jours pour le sang), sans que le virus ait pu cependant être cultivé, questionnant sur sa possible infectiosité. Les seules cultures qui se sont avérées positives ont concerné des prélèvements faits avant 30 jours, aucun au-delà, mais le nombre d’échantillons mis en culture est resté limité. L’analyse des échantillons a permis de mettre en évidence une décroissance progressive du taux de positivité par PCR, avec 61 % entre 14 et 30 jours, 43 % entre 1 et 2 mois, 21 % entre 2 et 3 mois, 6 à 7 % entre 3 et 5 mois, et 1 % au-delà de 5 mois, alors même qu’aucun prélèvement urinaire n’a été retrouvé positif au-delà de 60 jours. Les facteurs de risque identifiés d’excrétion virale prolongée dans le sperme ont été un âge plus élevé et un nombre plus faible d’éjaculations [21].

À l’inverse, chez les femmes, le taux de positivité des sécrétions vaginales reste bas et limité dans le temps : dans l’étude menée à Porto Rico en 2016-2017, seuls 2 prélèvements vaginaux sur 114 se sont avérés positifs, un (sur 2) fait avant le J7 du début des symptômes, et un (sur 112) fait au-delà de J60, aucun entre J7 et J60 [26]. Ces résultats sur des effectifs conséquents ont confirmé des données préliminaires observées chez des patientes espagnoles, où seul un prélèvement vaginal sur 4 avait été retrouvé positif avant 45 jours et aucun au-delà [30]. En revanche, une étude faite au Nicaragua en 2016-17 a retrouvé une excrétion virale dans les sécrétions vaginales bien plus fréquente et plus prolongée chez les femmes enceintes, avec une PCR positive sur les sécrétions vaginales chez 4 femmes sur 5, les 4 étant enceintes. L’excrétion virale maximale a été observée à 180 jours chez une patiente, sans qu’une culture n’ait été faite pour attester de l’infectiosité [29]. Cette infectiosité n’a en fait pu être mise en évidence qu’en phase aiguë virémique de l’infection chez la femme [27].

Cette différence d’infectiosité entre le sperme et les sécrétions vaginales joue d’ailleurs certainement un rôle supplémentaire dans le risque de contracter le virus Zika pour les partenaires de patients masculins infectés par le Zika. À ce titre, l’incidence presque deux fois plus importante du Zika chez les femmes de 15 à 65 ans à Rio de Janeiro par rapport aux hommes du même âge suggère cette contribution significative de la transmission sexuelle de l’homme vers la femme, une telle surincidence n’étant pas clairement observée pour le virus de la dengue [6].

Les points clés sur la transmission sexuelle sont résumés dans l’encadré 1.

Encadré I Points clés sur la transmission sexuelle

key facts about sexual transmission

- Cas index le plus souvent masculin, symptomatique ou asymptomatique.
- Rapport sexuel de tout type en cause : vaginal, oral, anal.
- Durée médiane de survenue du tableau clinique du cas secondaire rapporté au cas index = 13 jours (4-44 jours).
- Détection plus fréquente et plus prolongée du virus Zika dans le sperme par rapport aux sécrétions vaginales ouà la salive :
  - Sperme : maximum à 281 jours après le début des symptômes, mais rare au-delà de 5 mois, et associé à une infectiosité (culture positive) jusqu'à 69 jours après le début des symptômes [1, 21].
  - Sécrétions vaginales : en phase aiguë virémique (jusqu'à J7), exceptionnel au-delà, sauf peut-être chez les femmes enceintes (maximum de 180 jours), sans preuve d'infectiosité [26, 29].
- Transmission plus facilement observée en contexte de voyage en zone épidémique avec survenue d'une infection au virus Zika chez le ou la partenaire n'ayant pas voyagé.
- Rôle significatif de cette transmission sexuelle dans la surincidence d'infection Zika chez les femmes en âge de procréer dans les zones épidémiques.

## La prévention de la transmission sexuelle en zone d'endémie et chez les voyageurs

L’objectif prioritaire des mesures de prévention, en particulier sexuelle, vise à réduire le risque de malformations fœtales au cours de grossesses connues ou potentiellement à venir. À ce titre, les populations cibles principales de ces mesures sont les femmes enceintes, ayant un désir de grossesse ou en âge de procréer, ainsi que leur partenaire ou tout homme ayant une partenaire en âge de procréer, qu’il s’agisse de personnes vivant en zone épidémique ou de voyageurs.

Outre les mesures de prévention d’ordre sexuel, il est important de connaître et de respecter les mesures générales, valables pour toute la population, à savoir assurer une bonne protection personnelle anti-vectorielle diurne, consulter en cas de tableau clinique évocateur, tout en assurant une bonne connaissance sur l’infection à virus Zika et sur ses complications fœtales.

En zone d’endémie, chez les femmes enceintes, il convient d’éviter les relations sexuelles non protégées jusqu’à la fin de la grossesse [16]. Chez les femmes en âge de procréer ayant un désir de grossesse, il convient idéalement de reporter tout projet de grossesse pendant la durée de l’épidémie de Zika dans la zone où elles vivent ou d’envisager une contraception, mais aussi d’éviter tout rapport sexuel non protégé avec un partenaire ayant pu être infecté par le virus Zika [16]. Chez les hommes, il convient surtout d’éviter tout rapport sexuel non protégé avec une partenaire enceinte ou en âge de procréer pendant la durée de l’épidémie de Zika [16].

Chez les voyageurs, la situation est finalement assez superposable, en sachant que l’exposition, qu’elle soit vectorielle ou non, est potentiellement évitable, étant tributaire du voyage. Le risque n’existe, finalement, qu’en cas de voyage en zone de circulation virale.

Chez les femmes enceintes, quel que soit le terme, il convient d’éviter tout séjour en zone épidémique ou au moins de reporter le voyage après la grossesse. En cas de voyage, outre les mesures générales citées ci-dessus, il convient d’éviter tout rapport sexuel avec un homme infecté ou ayant pu l’être pendant toute la durée de la grossesse, de pratiquer un test sérologique 28 jours après le retour pour rechercher une éventuelle séroconversion, en l’absence de symptômes au retour et, en cas de positivité, de consulter en milieu gynécoobstétrical spécialisé [17].

Chez les femmes en âge de procréer, particulièrement en cas de désir de grossesse, les mesures sont assez similaires : il convient de reporter le séjour en cas de désir de grossesse, ou, en cas de voyage, de prendre une contraception orale ou de reporter la grossesse au moins 28 jours après le retour de voyage, tout en évitant tout rapport sexuel non protégé avec un homme infecté ou ayant pu l’être jusqu’à confirmation au retour de l’absence de risque de contamination du partenaire. Enfin, en cas de possibilité de grossesse, un test sérologique 28 jours après le retour pour rechercher une infection asymptomatique est utile [17].

Chez les hommes dont la partenaire serait en âge de procréer, il convient, en cas de voyage non évitable, d’éviter tout rapport sexuel non protégé avec une tierce personne infectée ou ayant pu l’être, et de pratiquer un dépistage sérologique à J28 du retour en cas de grossesse ou de désir de grossesse chez la partenaire, afin de documenter une séroconversion. Si celle-ci est observée (IgG +), la recherche de virus Zika par PCR dans le sperme à 2 occasions à au moins une semaine d’intervalle est nécessaire pour assurer l’absence de risque de transmission sexuelle : une telle transmission peut être exclue avec une probabilité satisfaisante si la sérologie est négative en IgG, bien que d’exceptionnels faux négatifs aient pu être observés avec des techniques ELISA de routine [9], ou, en cas de sérologie positive, si les 2 PCR successives sont négatives [17]. D’une façon générale, en l’absence de dépistage, il convient de maintenir des rapports sexuels protégés pendant une durée de 3 à 6 mois après une infection symptomatique ou après le retour d’une zone épidémique, en raison de la persistance prolongée d’une excrétion virale dans le sperme (Encadré 2).

Encadré 2 Prévention chez les voyageurs, si le voyage ne peut être évité

Prevention for travelers, if the trip cannot be avoided

- Mesures communes à tous les voyageurs :
  - Connaître les conséquences de l'infection au virus Zika, en particulier le risque d'embryofoetopathies.
  - Suivre une protection personnelle antivectorielle.
  - Consulter en cas de tableau clinique compatible avec une infection au virus Zika.
- Mesures spécifiques visant à limiter le risque de transmission aux femmes enceintes ou susceptibles de le devenir :
  - Limiter les voyages en zone à risque en cas de grossesse ou de désir de grossesse, ou préconiser une contraception efficace.
  - Rapports sexuels protégés pendant 3 à 6 mois au retour de voyage ou après une infection symptomatique au virus Zika chez l'homme.
  - Dépistage sérologique et éventuellement PCR chez l'homme en période épidémique, en cas de risque de contamination de la partenaire.

S’il paraît légitime de préconiser ces mesures en cas d’épidémie avérée, comme cela a été le cas en Amérique latine en 2016, ou en cas de circulation virale attestée par la présence de cas récents dans le pays visité, leur application paraît plus difficile à imposer pour un voyage dans une zone où les cas décrits, qu’ils soient sporadiques ou épidémiques, ne sont pas récents, le risque résiduel n’étant bien souvent pas connu. En définitive, seule une circulation active du virus paraît en effet être en mesure de servir de base à une telle recommandation, en sachant que cette « circulation active » est, dans le cas du virus Zika, difficile à établir, car imprévisible et peu visible. Le suivi du site de l’OMS ou du CDC américain peut être une aide pour évaluer ce risque épidémique, même s’il est actuellement compliqué d’avoir des données de circulation très récente [5].

## Conclusion

L’infection au virus Zika, bien que le plus souvent bénigne, expose à un risque significatif de malformations fœtales en cas de survenue chez une femme enceinte, même si ce dernier a été surévalué lors de l’épidémie de 2016 en Amérique latine. Sa transmission est essentiellement vectorielle, mais une transmission sexuelle est possible, particulièrement de l’homme vers la femme, où elle représente probablement une part non négligeable des cas chez les femmes en âge de procréer en zone d’endémie. Chez les voyageurs, des mesures de prévention sont possibles, d’autant plus faciles à préconiser que la situation du pays de destination est épidémique. Elles sont par contre plus difficiles à justifier en cas de voyage dans un territoire sans donnée épidémiologique récente.

## Liens d'intérêt

L’auteur ne déclare aucun conflit d’intérêts.
